# Alternative pacing strategies for optimal cardiac resynchronization therapy

**DOI:** 10.3389/fcvm.2022.923394

**Published:** 2022-09-27

**Authors:** Juan Hua, Qiling Kong, Qi Chen

**Affiliations:** Department of Cardiology, The Second Affiliated Hospital of Nanchang University, Nanchang, China

**Keywords:** cardiac resynchronization therapy, biventricular pacing, His bundle pacing, left bundle branch area pacing, leadless LV pacing, review

## Abstract

Cardiac resynchronization therapy (CRT) *via* biventricular pacing (BVP) improves morbidity, mortality, and quality of life, especially in subsets of patients with impaired cardiac function and wide QRS. However, the rate of unsuccessful or complicated left ventricular (LV) lead placement through coronary sinus is 5–7%, and the rate of “CRT non-response” is approximately 30%. These reasons have pushed physicians and engineers to collaborate to overcome the challenges of LV lead implantation. Thus, various alternatives to BVP have been proposed to improve CRT effectiveness. His bundle pacing (HBP) has been increasingly used by activating the His–Purkinje system but is constrained by challenging implantation, low success rates, high and often unstable thresholds, and low perception. Therefore, the concept of pacing a specialized conduction system distal to the His bundle to bypass the block region was proposed. Multiple clinical studies have demonstrated that left bundle branch area pacing (LBBAP) has comparable electrical resynchronization with HBP but is superior in terms of simpler operation, higher success rates, lower and stable capture thresholds, and higher perception. Despite their well-demonstrated effectiveness, the transvenous lead-related complications remain major limitations. Recently, leadless LV pacing has been developed and demonstrated effective for these challenging patient cohorts. This article focuses on the current state and latest progress in HBP, LBBAP, and leadless LV pacing as alternatives for failed or non-responsive conventional CRT as well as their limits and prospects.

## Introduction

Heart failure (HF) is a cardiovascular epidemic, with high morbidity and mortality and poor quality of life, especially in patients with HF and reduced ejection fraction (HFrEF) (1). According to the 2021 European HF guidelines, sodium–glucose cotransporter 2 inhibitors (SGLT2i) are used as a first-line therapy along with angiotensin-converting enzyme inhibitors (ACEi)/angiotensin receptor–neprilysin inhibitors (ARNI), β-blockers, and mineralocorticoid receptor antagonists (MRA) (2). However, the HF symptoms of some patients cannot be resolved, despite optimized medical treatments (OMT).

Cardiac resynchronization therapy (CRT) is a well-established modality that offers remarkable clinical benefits for patients with medically refractory HF (3). It has a class IA indication for symptomatic HF patients with sinus rhythm (SR), a QRS ≥ 150 ms, left bundle branch block (LBBB) QRS morphology, and a left ventricular ejection fraction (LVEF) ≤ 35%, despite OMT according to the 2021 European Society of Cardiology (ESC) guidelines on CRT (4). Conventional CRT *via* biventricular pacing (BVP) is non-physiological with the fusion of the epicardial LV wavefront and the endocardial wavefront from the right ventricular (RV) apex, leaving some degree of dyssynchrony. However, conventional CRT is precluded in a proportion of eligible candidates due to anatomic or technical constraints such as occluded venous access, an inappropriate coronary sinus (CS) anatomy, or a high threshold in regions of fibrosis (5, 6). In addition, approximately 30% of recipients are non-responsive to CRT due to the inability to effectively stimulate diseased tissue, or suboptimal LV lead placement (7, 8).

For these reasons, physicians and engineers have been working together to overcome the challenges of LV lead implantation but have also shown increased interest in developing physiological pacing techniques to improve CRT effectiveness. His bundle pacing (HBP) has increased in use by activating the His bundle but is restricted by implant challenges, low success rates, and a high and often unstable pacing threshold (9–12). Therefore, the concept of pacing the specialized conduction system distal to the His bundle to bypass the block region has been introduced (13). Multiple clinical studies reported that left bundle branch area pacing (LBBAP) has electrical resynchronization that is comparable with that of HBP but superior due to its simpler operation, higher success rate, and low and stable pacing threshold (13–17). Despite their well-demonstrated effectiveness, the resulting complications of transvenous leads and typical pocket infections remain a non-negligible limitation (18, 19). Thus, leadless cardiac pacing has been engineered and demonstrated as having potential efficacy for treating those challenging patient cohorts (20, 21).

**FIGURE 1:**
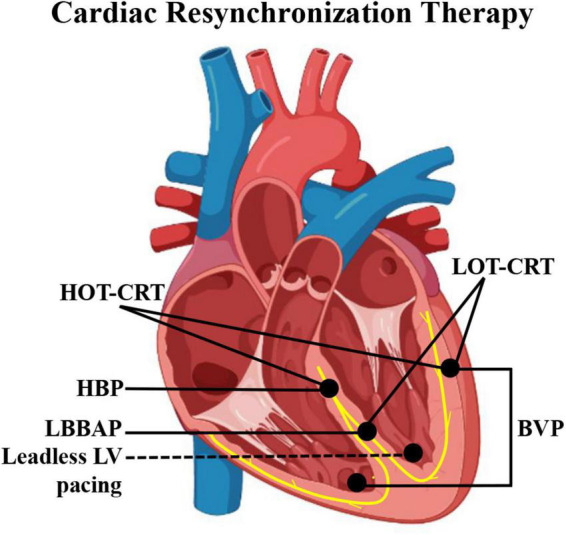
Schematic diagram of pacing electrode positions of different CRT modalities. BVP, biventricular pacing; CRT, cardiac resynchronization therapy; HBP, His bundle pacing; HOT-CRT, His-optimized CRT; LBBAP, left bundle branch area pacing; LOT-CRT, LBBAP-optimized CRT.

From CS epicardial pacing to leadless endocardial stimulation, various LV pacing alternatives reportedly improve CRT effectiveness (Figure 1). This review focuses on the current state and latest progress of HBP, LBBAP, and leadless LV pacing as alternatives for impossible or failed conventional CRT as well as their limits and future areas of improvement.

## Benefits and limits of biventricular pacing-cardiac resynchronization therapy

The efficacy of CRT in patients with HF has been demonstrated in numerous trials. The Multisite Stimulation in Cardiomyopathies (MUSTIC) study was the first to assess the clinical outcomes of CRT in 67 patients with severe HF (22). Finally, 48 patients completed both phases of the study. The quality-of-life score improved by 32%, while hospitalizations decreased by 67.7%. Similarly, the Multicenter InSync Randomized Clinical Evaluation (MIRACLE) trial was the first double-blind trial to assess the CRT outcomes in 453 patients with moderate to severe HF with an LVEF ≤ 35% and QRS ≥ 130 ms (23). CRT improved the New York Heart Association (NYHA) class, quality of life, and LVEF and reduced hospitalization and intravenous interventions. The Cardiac Resynchronization–Heart Failure (CARE-HF) trial was mainly conducted to assess the morbidity and mortality of CRT in 813 patients with HF (NYHA class III/IV) (24). The results indicated that CRT reduced mortality of any cause, increased the LVEF, and improved symptoms and quality of life. Subsequently, the REVERSE, MADIT-CRT, and RAFT trials assessed the efficacy of CRT in mildly symptomatic HF (25–27). These results showed that CRT significantly delayed the time to the first HF hospital stay or death, reduced the risk of HF events, and improved LV reverse remodeling.

However, approximately 30% of candidates respond unfavorably to CRT or even worsen (23, 24, 28). In fact, the optimal LV pacing site is not always consistent with the right branch of the CS. In addition, phrenic stimulation, occlusion of the CS anatomy, or other anatomical constraints would hamper the procedure. Thus, several approaches based on BVP-CRT, such as the adaptive CRT algorithm (29), the SyncAV algorithm (30), and multipoint pacing (MPP) (31), have improved the effectiveness of BVP-CRT. Notably, the optimal electrode position was not equal to that of the optimal treatment. In addition, little experience has been gained regarding the optimal programming of pacemakers after MPP. Furthermore, the higher battery use of MPP also prevents its recommendation.

## Alternative pacing strategies for conventional cardiac resynchronization therapy

Despite great advances in multipolar LV electrodes and MPP, non-response due to suboptimal lead position remains a critical problem. To overcome the challenges of the CS approach, physicians have strived for alternative solutions, such as surgical LV epicardial lead placement, LV endocardial pacing, or HBP to most recently leadless LV pacing. However, surgical LV epicardial lead placement does not always deliver additional improvement in the LVEF versus conventional CRT, and it is inherently more invasive and challenging in patients with a previous history of heart surgery (32). Moreover, the greatest concern for LV endocardial pacing is the risk of thromboembolic complications and the need for lifelong anticoagulation (33). By contrast, physiological pacing modalities have advantages over conventional LV epicardial and endocardial pacing. Next, this review focuses on the progress, limits, and prospects of HBP, LBBAP, and leadless LV pacing used for CRT.

## His bundle pacing for cardiac resynchronization therapy

His bundle pacing activates the His–Purkinje system (HPS) and restores physiological activation of the ventricles. HBP is defined as the presence or absence of His–Purkinje conduction disease (HPCD) according to four basic criteria (34): (1) relationship between the His-QRS (H-QRS) and stimulus-QRS (S-QRS) intervals; (2) the presence or absence of direct capture of the local ventricular electrogram (EGM) on the pacing lead; (3) QRS duration (QRSd) and morphology; and (4) capture thresholds. Broadly, there are two forms of HBP: selective HBP (S-HBP), in which the His bundle is exclusively captured; and non-selective HBP (NS-HBP), in which both the His bundle and its surrounding ventricular tissues are captured. The form of S-HBP or NS-HBP is usually dependent on the location of the pacing electrode in relation to the His bundle and the surrounding tissue (35). S-HBP can be differentiated from NS-HBP by review of the His bundle EGM on the pacing lead (34, 36): the local ventricular EGM is discrete and separate from the pacing stimulus on S-HBP, whereas it is fused with the pacing stimulus on NS-HBP. The specific criteria for S-HBP or NS-HBP are as follows (9, 34):

**S-HBP**: (1) S-QRS = H-QRS with an isoelectric interval. In patients with HPCD, S-QRS ≤ H-QRS with BBB correction and S-QRS ≤ or > H-QRS without BBB correction; (2) discrete local ventricular EGM in HBP leads with the stimulus to the local ventricle (S-V) = His to the local ventricle (H-V); (3) paced QRS = native QRS. In patients with HPCD, paced QRS < native QRS with BBB correction; paced QRS = native QRS without BBB correction; and 4) a single capture threshold (His capture) was observed. In patients with HPCD, two distinct His capture thresholds (with and without BBB correction) may be observed.

**NS-HBP:** (1) S-QRS < H-QRS (usually 0, S-QRS_end_ = H-QRS_end_) with or without an isoelectric interval. In patients with HPCD, S-QRS_end_ < H-QRS_end_ with BBB correction; (2) direct capture of local ventricular EGM in HBP lead by a stimulus artifact; (3) paced QRS > native QRS. In patients with HPCD, paced QRS ≤ native QRS with BBB correction and paced QRS > native QRS without BBB correction; (4) usually, two distinct capture thresholds (His bundle capture and RV capture) are observed. In patients with HPCD, three distinct capture thresholds (with or without correction for BBB and RV capture) may be observed.

Theoretically, S-HBP may be advantageous over NS-HBP in terms of clinical outcomes. Instead, few hemodynamic and clinical differences were observed between these two forms of capture, probably owing to the rapid conduction of the HPS relative to the ventricular myocardial conduction (37, 38).

### Evidence for resynchronization using His bundle pacing

In 2000, HBP was first described in 12 HF patients with atrial fibrillation, cardiomyopathy, and improvements in LV function after HBP and atrioventricular node ablation (39). In 2012, a series of direct HBP (DHBP) was reported in 16 patients after CS approach failure (40). Of these, LBBB was corrected in 13 of 16 patients, and permanent HBP (pHBP) was performed successfully in nine of 13 patients. LBBB was corrected using pHBP, with a mean QRS reduction (166 ± 8 to 97 ± 9 ms; *P* < 0.01). Subsequently, a prospective study assessed the HBP outcomes of patients with HF and LBBB (18). A total of 74 patients were enrolled, and pHBP was successful in 56 of them (75.7%). Of them, 30 had completed a 3-year follow-up with an increased mean LVEF (32.4 ± 8.9% to 55.9 ± 10.7%, *P* < 0.001) and decreased LV end-systolic volume (LVESV; 137.9 ± 64.1 to 52.4 ± 32.6 mL, *P* < 0.001). Similarly, the outcomes of HBP were explored in 106 CRT-eligible or -failed patients (41). Among them, HBP was successful in 95 patients (89.6%). During an average follow-up of 14 months, it also delivered significant QRS narrowing, increased LVEF, and improved NYHA class. Lead-related complications were observed in seven patients. These studies demonstrated that HBP may be a promising treatment for failed BVP.

### His bundle pacing versus biventricular pacing for cardiac resynchronization therapy

Multiple studies demonstrated that HBP may be an effective alternative to BVP; however, whether it is equal to or better than BVP requires further evaluation. The first crossover study to compare the outcomes of HBP and BVP enrolled 29 patients for HBP as an alternative to BVP (10). Finally, 21 of 29 patients achieved narrow-paced QRSd. The baseline LVEF was 26% with improvements at 6 months in the HBP (32%) and BVP (31%). A similar result of HBP delivering a greater reduction in QRSd, LV activation time (LVAT), and LV dyssynchrony index (LVDI) than BVP was reported (42). The His-SYNC trial was the first randomized comparison of HBP and BVP using treatment-received (TR) and per-protocol (PP) analyses (43). A total of 41 patients were enrolled and randomized into HBP (*n* = 21) and BVP (*n* = 20) groups. Compared with BVP, HBP achieved a narrower mean QRSd, regardless of TR or PP analyses (TR: 125 ± 22 vs. 164 ± 25 ms, *P* < 0.001; PP: 124 ± 19 vs. 162 ± 24 ms, *P* < 0.001). Furthermore, a non-significant trend toward a higher echocardiographic response was observed. There were also no significant intergroup differences in CV hospitalization and mortality. Another randomized trial of HBP versus BVP (His-Alternative) was performed in patients with symptomatic HF and LBBB (44). The pacing thresholds of HBP were higher than those of BVP, both at implantation and at the 6-month follow-up. Using PP analysis, the LVEF was significantly increased, and the 6-month LVESV was lower in patients with HBP than in those with BVP. These data revealed that HBP was equivalent to, or even better than, BVP in some cases; however, further investigations are required to confirm these findings.

### His-optimized cardiac resynchronization therapy

The use of HBP alone may not always be optimal for obtaining QRS narrowing. Several studies have explored whether CRT could maximize electrical resynchronization by HBP fused with sequential LV pacing, termed His-optimized CRT (HOT-CRT) (45–48). HOT-CRT was attempted in 27 patients with LBBB/intraventricular conduction defect (IVCD) partially corrected by HBP alone (45). HOT-CRT produced a greater narrowing of the mean QRSd to 120 ± 16 ms (vs. baseline, BVP, or HBP; *P* < 0.0001). LVEF improved significantly (from 24 ± 7% to 38 ± 10%, *P* = 0.001) after a mean follow-up of 14 ± 10 months. Similar results with a narrower QRSd, increased LVEF, and improved NYHA were reported in other studies (46–48). In addition, HOT-CRT versus HBP resulted in significant QRS narrowing, thus achieving electrical resynchronization in four of five patients with IVCD (45). These data indicate that HOT-CRT produced more pronounced QRS narrowing and improved clinical outcomes than HBP alone. Particularly, HOT-CRT could further optimize electrical resynchronization in patients with advanced cardiomyopathy and conduction disease; however, this finding requires further verification.

### Limits of His bundle pacing

These trials indicated that HBP generates a narrow paced QRS and improves clinical outcomes, which seem potential for CRT. However, HBP has some limitations. First, the major limit is the inability to map the precise location of the His bundle, which is approximately 1–2 mm in diameter (49). The mean success rate of HBP was approximately 79.8% (mostly were performed with the SelectSecure 3830 lead; Medtronic, Minneapolis, MN, United States), while the lead-related complication rate was 6% (50). Second, 30–40% of LBBB cannot be corrected by HBP because of the presence of lesions distal or more extensive to the conduction tract during implantation (51). Third, the HBP threshold increased over time. About 53.6% of patients had a significant increase in the His capture threshold after a mean follow-up of 3 years (18). A progressive increase in the pacing threshold implies a shortened battery longevity. HBP may also undersense the ventricle and oversense the atrium, thus resulting in crosstalk. Finally, most of the current research conclusions on the application of HBP in HF with LBBB were derived from the data of HBP with failed BVP, while large-scale randomized controlled clinical trials of HBP and BVP are lacking. Furthermore, the number of patients who responded to HBP and did not respond to BVP was small, and similarly, the number of patients who included HOT-CRT with IVCD HF and were refractory to BVP or HBP was small.

## Left bundle branch area pacing for cardiac resynchronization therapy

In 2017, Huang et al. (13) first introduced left bundle branch pacing (LBBP) in a patient with HF and LBBB and confirmed its feasibility and safety. LBBP captures the proximal LBB or its branches with or without the LV septal myocardium. LV septal pacing (LVSP) exclusively captures the LV septal myocardium. However, during the early stage of LBBP, the criteria for LBB capture are not well defined and uniform. With the increased use and further research on LBBP, the definition of LBB capture is gradually becoming definitive (52). Broadly, there are two forms of LBBP: selective LBBP (S-LBBP) exclusively captures the LBB, whereas non-selective LBBP (NS-LBBP) captures the LBB along with the surrounding local myocardium. The detailed characteristics of LBBP are defined as follows: (1) RBBB pattern, (2) LBB potential, (3) S-LBBP with specific ECG changes and a discrete component on EGM, and (4) a constant and shortest stimulus to the LVAT, regardless of high or low pacing outputs. LBBP is differentiated from LVSP based on the mentioned characteristics of the indirect criteria for LBB capture. Thus, LVSP is mistakenly considered LBBP in some cases. Wu et al. (53) proposed retrograde His bundle potential or anterograde left conduction system potentials to directly confirm LBB capture, which can more accurately distinguish between LBBP and LVSP. However, this method is complicated and unsuitable for routine clinical use. In this context, the concept of LBB area pacing (LBBAP) has been proposed, that is, LBBP or LVSP, without clear evidence for LBB capture (4). During LBBAP, the QRS morphologies in lead V1 are typically demonstrated as Qr (60.7%), qR (19.6%), rSR’ (7.1%), or QS (12.5%) patterns, and the duration of the terminal R’ wave was significantly shorter than that of native RBBB (54).

### Evidence for resynchronization using left bundle branch area pacing

Several single-center studies with short follow-up periods have confirmed the potential of LBBAP in patients with HF and wide QRS (13, 55). A prospective multicenter medium-term study assessed LBBP in patients with LBBB and non-ischemic cardiomyopathy (56). LBBP was successful in 61 of 63 patients (97%). It produced a shortened mean QRSd (169 ± 16 to 118 ± 12 ms, *P* < 0.001), increased LVEF (33 ± 8% to 55 ± 10%, *P* < 0.001), decreased LVESV (123 ± 61 to 67 ± 39 mL, *P* < 0.001), and improved NYHA class (2.8 ± 0.6 to 1.4 ± 0.6, *P* < 0.001). A subsequent long-term trial with a larger sample size (*N* = 632) assessed LBBP feasibility and safety (57). LBBP was successful in 618 (97.8%) patients, and the mean follow-up was 18.6 ± 6.7 months. A significant decrease in QRSd was observed in patients with LBBB. LVEF after LBBP improved in patients with QRS ≥ 120 ms (*N* = 88). No serious complications occurred during the procedure or follow-up. A similar result was reported by another large study (*N* = 325) (19) in which LBBAP was successfully achieved in 277 (85%) patients. During a mean follow-up period of 6 ± 5 months, LBBAP also resulted in significant QRS narrowing and improved LVEF. In a current meta-analysis, LBBP for CRT resulted in a narrower QRSd and an increased LVEF than baseline (58). Nonetheless, relatively few studies have examined LVSP for CRT. To date, LVSP has been demonstrated to generate short-term hemodynamic improvement and electrical resynchronization equal to that of BVP and possibly HBP (59). These data demonstrated that LBBAP may be a promising rescue strategy for failed BVP; however, further investigations are needed.

### Left bundle branch area pacing versus biventricular pacing for cardiac resynchronization therapy

Several clinical trials have explored whether LBBAP is equal or superior to conventional CRT (15–17, 60, 61). In these studies, LBBAP/LBBP produced a narrower paced QRSd than did BVP as expected. Accordingly, LBBAP/LBBP resulted in an increased LVEF and improved NYHA class. LBBAP improved the LVEF more than BVP in this study (16). By contrast, LBBAP/LBBP was equivalent to BVP in other studies (15, 17, 61). LBBP, HBP, and BVP were compared in 137 non-randomized patients with an LVEF ≤ 40% and typical LBBB (60). Finally, HBP and LBBP delivered similar improvement in the LVEF and NYHA class after the 1-year follow-up, which was significantly higher than that in BVP. Furthermore, some meta-analyses of LBBAP for CRT have been reported (62, 63). A meta-analysis compared LBBAP and BVP for CRT (62). Compared with BVP, LBBAP produced significantly narrower QRSd with a mean difference (MD) 29.18 ms, LVEF improvement of 6.93%, LVEDD reduction of 2.96 mm, and NYHA class improvement of 0.54. Similarly, a network meta-analysis compared LBBAP, HBP, and BVP for patients requiring CRT (63). Compared with BVP, LBBAP produced greater LVEF improvement with an MD of 7.17%, followed by an HBP of 4.06%. In addition, HBP produced a narrower QRSd with an MD of 31.58 ms, followed by an LBBAP of 27.40 ms. There were no differences in LVEF improvement and QRS narrowing for LBBAP versus HBP. These data indicated that LBBAP, comparable with HBP, may be superior to BVP, but further evaluations are needed.

### Left bundle branch area pacing-optimized cardiac resynchronization therapy

To our knowledge, proximal LBB pacing is inherently limited by its inability to restore physiological activation of the lateral wall of the LV in patients with a distal conduction delay (19). Thus, it may not always be optimal for QRS narrowing by LBBAP alone. Whether LBBAP-optimized CRT (LOT-CRT), LBBAP combined with CS LV pacing, would be advantageous over LBBAP or BVP is unknown. Thus, the LOT-CRT was assessed in an international multicenter study of non-consecutive patients who were indicated for CRT or non-responders (64). LOT-CRT was successful in 91 of 112 patients (81%). The average follow-up was 7.8 ± 2.3 months. LOT-CRT generated significantly greater narrowing of QRSd to 144 ± 22 ms (vs. baseline, BVP, and LBBAP, *P* < 0.0001), increased LVEF (28.5 ± 9.9% to 37.2 ± 12%, *P* < 0.0001), and decreased LVEDD (62.0 ± 8.9 to 59.1 ± 9.1 mm, *P* < 0.0442) and *N*-terminal pro-hormone B-type natriuretic peptide (5,668 ± 8,249 to 2,561 ± 3,555 pg/mL, *P* < 0.0001). These results indicated that LOT-CRT provided greater electrical resynchronization and clinical benefits than BVP or LBBAP alone, but further research is needed to confirm this finding.

### Limits of left bundle branch area pacing

Multiple studies demonstrated the technical advantages and clinical potential of LBBAP. It has comparable LV synchrony with HBP but a high success rate of 81.1–97% (15, 16, 19, 56, 65) and a low lead-related complication rate of 1.5% (65) with the SelectSecure 3830 lead (Medtronic, Minneapolis, MN, United States). Currently, other stylet-driven conventional active fixation pacing leads can also effectively obtain LBBAP, such as the Solia S60 lead (Biotronik, SE & Co., KG, Germany) (66–68), the Ingevity pacing lead (Boston Scientific Inc., Marlborough, MA, United States) (68), and the Tendril 2088TC lead (Abbott, Inc., United States) (68, 69). In addition, LBBAP has a lower and stable threshold and high perception, and it is preferred for patients with a block far beyond the His bundle branch. Furthermore, the broad and expansive nature of LBB makes LBBAP implantation simpler and faster than that of HBP (70). However, some issues should still be noted, including its acute and long-term safety. Several complications may occur during the procedure, such as LV perforation as the lead advances into the deep interventricular septum (IVS) (71). Thus, a pre-procedural IVS thickness evaluation would be safer. Furthermore, the lead should be rapidly rotated until it penetrated deep into IVS, and fluoroscopic image and pacing parameters and morphologies should be monitored to avoid the perforation of IVS during the process (13, 19). In addition, the safety of postoperative lead extraction after a long duration has been the focus of much attention. Chen et al. (72) reported that three of 612 patients repositioned the lead during the follow-up (one postoperative septum perforation and one postoperative lead dislodgement at 1 month, and one postoperative lead dislodgement at 1 month after repositioned for 5 months). These leads were extracted and repositioned at different sites, and the parameters were stable at an additional 1-year follow-up. Similarly, Su et al. (57) reported a septal perforation during the follow-up in one patient, and the lead was removed and reimplanted without serious complications. Collectively, further research is needed to firmly establish the safety of LBBAP for CRT, particularly for lead extraction over a long duration after the procedure.

## Leadless left ventricular pacing: New direction for patients after coronary sinus approach failure

Despite the well-demonstrated effects of HBP and LBBAP, transvenous leads and typical pocket infections remain a non-negligible limitation (18, 19). Leadless cardiac pacing has been proposed to address these complications. The WiSE-CRT system (EBR Systems Inc., Sunnyvale, CA, United States) is the only currently available leadless LV pacing system that comprises a subcutaneous pulse generator transmitter and LV endocardial receiver electrode (73). In this system, acoustic energy is converted from the pulse generator transmitter, located subcutaneously at the fourth, fifth, or sixth intercostal space, to electrical stimulation of a receiver electrode implanted into the LV cavity. The system works in conjunction with a co-implant of RV pacing, which could be a conventional device such as a pacemaker or implantable cardioverter defibrillator (ICD) or a leadless pacemaker such as Micra (Medtronic, Minneapolis, MN, United States). Biventricular pacing is accomplished by perceiving the RV pacing output of the co-implant, followed by the system immediately transducing acoustic energy to electrical stimulation of the LV electrode, thus achieving near-synchronous RV and LV pacing.

### Evidence for leadless pacing

In 2014, the Wireless Stimulation Endocardially for CRT (WiSE-CRT) study (20) included 17 HF patients, two-thirds of whom showed ≥ 1 NYHA class improvement at the 6-month follow-up. The Safety and Performance of Electrodes implanted in the Left Ventricle (SELECT-LV), a prospective multicenter non-randomized trial, enrolled 35 CRT-indicated patients who “failed” conventional CRT and underwent implantation of leadless pacing (21). The procedure was successfully performed in 34 (97.1%) patients. Of them, 84.8% (*N* = 28) showed an improved clinical composite score and 66% (*N* = 21) gained a ≥ 5% absolute increase in the LVEF at 6 months. Of note, serious procedure/device-related complications were observed in 8.6% of patients (*N* = 3) within 24 h and 22.9% of patients (*N* = 8) between 24 h and 1 month. A real-world experience with the WiSE-CRT system was shared in an international trial (ClinicalTrials.gov identifier: NCT02610673) (74) in which procedural success and the delivery of biventricular endo-pacing occurred in 85 of 90 patients (94.4%). The acute (within 24 h), 1- to 30-day, and 1- to 6-month complication rates were 4.4% (*N* = 4), 18.8% (*N* = 17), and 6.7% (*N* = 6), respectively. A total of five deaths (5.6%) occurred within 6 months. HF symptoms improved in 70% of patients. Subsequently, the Stimulation of the Left Ventricular Endocardium for Cardiac Resynchronization Therapy (SOLVE-CRT) trial assessed the short-term outcomes of the WiSE-CRT system in cases without prior implant experience (75). WiSE-CRT was successful in all 31 patients. Of them, 30 completed the 6-month follow-up. In total, 14 (46.7%) patients achieved ≥ 1 NYHA class improvements and an improved LVEF, decreased LVESV, and increased LV end-diastolic volume (LVEDV); three (9.7%) device-related complications occurred: insufficient LV pacing (*N* = 1), embolization of an unanchored LV electrode (*N* = 1), and skin infection (*N* = 1). These results indicated that biventricular endo-pacing from the WiSE-CRT system was effective in cases of failed conventional CRT or non-response, but complications must be noted.

### Totally leadless cardiac resynchronization therapy

The aforementioned trials of leadless LV endocardial pacing were combined with a traditional pacemaker or ICD instead of a totally leadless CRT. The successful coexistence of Micra and the WISE-CRT system was first reported in 2019 (76). Also, two other cases were published in the same year or later (77, 78). The patients in these case reports have a common characteristic, that is, they have a complex history including old age, infection, valvular replacement surgery, or venous occlusion. These patients achieved a narrower QRSd and satisfactory clinical outcomes without serious complications after leadless CRT. These cases raised the possibility of completely leadless CRT. Subsequently, multiple European centers shared their experiences with totally leadless CRT (79). A total of eight patients from six centers underwent combination treatment with Micra and WiSE-CRT systems. The QRSd reduction immediately after WiSE-CRT implantation was significant (204.38 ± 30.26, 137.5 ± 24.75 ms, *P* = 0.012), and it was maintained at the 6-month follow-up. Only a significant improvement in the LVEF was achieved after WiSE-CRT implantation (28.43 ± 8.01% vs. 39.71 ± 11.89%, *P* = 0.018) without evidence of LV reverse remodeling and improved NYHA class. A current meta-analysis of leadless LV pacing for CRT (80) included five studies (four with RV leads of conventional devices and one with Micra) involving 181 total patients in the final analysis. The success rate of the procedure was 90.6%. It generated a mean increase in the LVEF with an MD of 6.3% and NYHA class improvement of 0.43. Notably, the procedure-related complications and mortality rates were 23.8% and 2.8%, respectively. However, this new pacing modality was used in only a small number of patients, and further studies are needed to confirm its feasibility and safety.

### Limits of leadless left ventricular pacing

Taken together, these data support the efficacy of leadless LV pacing as an alternative in patients in whom CRT is impossible or ineffective. It significantly reduces diaphragm stimulation, avoids mitral regurgitation, and can be performed at multiple physiological pacing positions. In addition, the receiver electrode was completely endothelialized for approximately 4 weeks; therefore, long-term anticoagulation was not required (74). However, leadless LV pacing has several limitations. First, it is challenging to choose a suitable acoustic window (distance < 10 cm and angulation < 30°) to effectively transmit ultrasound. Second, some regions of the left lateral free wall of the enlarged LV may be difficult to reach owing to the current delivery sheath. Third, the battery life projections averaged 18 months (range, 9–42 months) (20), which is often overestimated and should be improved. Moreover, the procedure is complex and has a relatively high complication rate. However, security issues are a common problem in the early stages of any novel technique. Improvements in the safety profile, such as different delivery sheaths, increased operator experience, and practice modifications, would reduce its complication rates and increase its widespread use. Additionally, pre-procedural cardiac computed tomography can be used to identify the optimal positioning of the receiver electrode based on indicators such as scar burden, simulated latest activation (81), and hemodynamic assessment (82).

## Conclusion

In summary, HBP, LBBAP, and leadless LV pacing have been demonstrated as potential alternatives for optimal CRT when conventional CRT fails. Each technique has its advantages and disadvantages (Table 1). HBP and LBBAP have shown more effective electrical resynchronization than conventional BVP. Accordingly, they provided equivalent or even superior clinical outcomes in some challenging cohorts. However, transvenous leads remain a major limitation of these pacing modalities. Thus, leadless LV pacing has been developed and demonstrated to provide more physiological LV endocardial activation coupled with clinical benefits. Furthermore, the advantage of leadless LV pacing would become more pronounced in cases of venous occlusion or lead infection. With a better understanding of HBP, LBBAP, leadless LV pacing, and their appropriate candidates, it is more likely that the most suitable alternative will be chosen when conventional CRT is impossible or ineffective.

**TABLE 1:** Comparison of BVP, HBP, LBBAP, and leadless LV pacing.

	BVP	HBP	LBBAP	Leadless LV pacing
Since (year)	1990	2000	2017	2014
Lead	LV lead, RV lead, (RA lead)	His lead, (RA lead)	LBB lead, (RA lead)	RV lead/none
LV or His or LBB lead position	CS	Proximal to His-bundle or in the His-bundle	Distal to His-bundle	Into the LV cavity
LV or His or LBB lead threshold	Generally high (15–17, 60)	Generally high and unstable (43, 44, 60)	Generally lower and stable (13–17, 60)	Generally high (20, 78)
Stim-LVAT	Mildly shortened	Significantly shortened	LBBP: shortest and constant LVSP: longer than LBBP	Theoretically near normal
Implant success rate	92.4%∼97% (23–25)	79.8% (50)	81.1%∼97% (15, 16, 19, 56, 65)	90.6% (80)
ΔLVEF	+ 3.7%∼5.9% (23–25)	+10.87∼14.32% (50)	+ 14.31∼ 22.69% (58)	+4.35∼8.19% (80)
ΔQRSd	−20∼−12 ms (23)	−50.67∼−36.34 ms (50)	−61.64∼−53.72 ms (58)	−67∼−27.3 ms (21, 79)
Procedure-related complication rate	6.1∼12.6% (23–25)	6% (50)	1.5% (65)	23.8% (80)
Battery life	5–6.5 years	Comparable to BVP	Relative longer than HBP	Mean of 18 months (9–42 months)
Advantages	Conventional approach with high level of evidence, well managed technique	Physiological stimulation, narrower paced QRSd	Physiological stimulation, narrower paced QRSd, low and stable threshold	No transvenous lead, endocardial pacing, no need for long-term anticoagulation
Disadvantages	Electrical constraint, high threshold, limited location possibility, phrenic stimulation	Transvenous lead, high threshold, technical and challenging procedure	Risk of IVS perforation, long-term safety and lead extraction need further evaluated	Recent technique with little evidence, need for an acoustic window, complex procedure

BVP, biventricular pacing; CS, coronary sinus; HBP, His bundle pacing; LBBAP, left bundle branch area pacing; LBBP, left bundle branch pacing; LV, left ventricular; LVEF, left ventricular ejection fraction; LVSP, left ventricular septal pacing; QRSd, QRS duration; RA, right atrium; RV, right ventricle; stim-LVAT, stimulus to left ventricular activation time. Δ represents an absolute increase from baseline after pacing.

## Author contributions

JH conducted the reference analysis and wrote the manuscript. QK contributed to the reference collection and manuscript revision. QC contributed to the topic conception, manuscript revision, and decided to submit for publication. All authors contributed to the article and approved the submitted version.
